# Nail Melatonin Content: A Suitable Non-Invasive Marker of Melatonin Production

**DOI:** 10.3390/ijms22020921

**Published:** 2021-01-18

**Authors:** Alex Gomez-Gomez, Blanca Montero-San-Martin, Noemí Haro, Oscar J. Pozo

**Affiliations:** 1 Integrative Pharmacology and Systems Neuroscience Group, Institut de l’Hospital del Mar d’Investigacions Mèdiques (IMIM), Doctor Aiguader 88, 08003 Barcelona, Spain; agomez@imim.es (A.G.-G.); nharo@imim.es (N.H.); 2 Department of Experimental and Health Sciences, University Pompeu Fabra (CEXS-UPF), Doctor Aiguader 88, 08003 Barcelona, Spain; 3 Laboratory Medicine, La Paz University Hospital, Paseo de la Castellana 261, 28046 Madrid, Spain; blancamontero1912@gmail.com

**Keywords:** melatonin, nails, fingernails, aging, liquid chromatography, mass spectrometry

## Abstract

Melatonin plays multiple physiological roles in the human body. Evaluation of melatonin production by the determination of urinary 6-sulfatoxymelatonin in 24-h samples has important drawbacks which hinder the successful evaluation of melatonin production in large cohorts. Here, we evaluated the potential of nail analysis for estimating melatonin production. Firstly, mass spectrometry methodology for the determination of melatonin in nails was optimized and successfully validated. The method was found to be linear in the range 6.5–830 fg/mg with intraday and interday accuracy in the range 100–104 %, precision below 15 % and a LOD of 3.5 fg/mg. Secondly, nail melatonin concentrations from 84 volunteers (age 5–96) were determined. The expected correlation between melatonin and age was obtained (correlation coefficient −0.615; *p* < 0.001). Additionally, we showed that fingernails are preferable to toenails to determine nail melatonin content. Finally, fingernails collected for 180 days after melatonin administration (two volunteers, 1.9 mg/night during 5 days) were analyzed. Nail melatonin concentrations immediately rose after administration and went back to pre-administration values after ≈100 days in both volunteers. Our results suggest that melatonin determination in nails is a suitable non-invasive tool for the estimation of global melatonin production. Due to the easy collection and storage of nails, the long-term information obtained and the multiple functions of melatonin, nail melatonin content might complement dim light melatonin onset, which is commonly measured from plasma/saliva samples, paving the way for melatonin research.

## 1. Introduction

Melatonin is a hormone mainly released by the pineal gland at nighttime [1]. The main role of melatonin is the regulation of the sleep–wake cycle, although it plays additional roles, such as being an antioxidant by stimulating the synthesis of glutathione [2], and by scavenging hydroxyl and peroxyl radicals and stimulating superoxide dismutase, glutathione peroxidase and catalase [3,4]. Other functions of melatonin are related to body weight regulation [5], immune system maintenance [6] and reproduction [7,8].

Melatonin underproduction has been related to different types of dementia [9,10], severe pain [11], cancer [12,13], diabetes type 2 [14] and other diseases [15,16,17,18]. Melatonin synthesis is also highly related to age. The levels of melatonin are decreased gradually over the life-span and are directly related to lowered sleep efficacy [19,20]. In fact, some of melatonin’s actions have been reported to be beneficial for the process of aging [21,22,23].

Due to the important roles of melatonin, suitable strategies able to evaluate melatonin production are required. Melatonin determination presents important analytical challenges. Thus, researchers must measure levels at the femtomole range in the presence of compounds of similar molecular structure in complex matrices. Poor methodologies can imply the publication of studies that are seriously flawed [24,25].

The proper interpretation of the results is as important as the reliable analytical determination. Thus, single-point determinations of melatonin in either saliva or plasma are meaningless, and their use should be avoided [26]. Melatonin determination in serial plasma/saliva samples is preferred. This approach is commonly used to obtain the dim light melatonin onset (DLMO), the most accurate marker for assessing the circadian pacemaker [1]. The estimation of the global production of melatonin is commonly performed by the determination of urinary 6-sulfatoxymelatonin in 24-h samples [27]. Although this approach has been shown to be useful in controlled scenarios [28,29], its practical application to studies based on large cohorts of volunteers is limited by the tediousness of sample self-collection. Additionally, the 24-h concentration of urinary 6-sulfatoxymelatonin only provides information about the melatonin production in the last day.

Therefore, new alternatives are required in order to establish a global production of melatonin, especially in large-cohort studies. A potential option might be the determination of melatonin in other matrices such as nails. Nails provide a unique feature. At a grow rate around 3 mm/month [30], fingernail samples can provide long-term information with the simplest sampling and storage. Nails have already been used for the evaluation of chronic stress by successfully correlating nail cortisol and the perceived stress scale [31]. Additionally, the usefulness of nails to determine the administration of drugs of abuse, e.g., benzodiazepines, tetrahydrocannabinol or cocaine, has also been demonstrated [32]. Therefore, the potential determination of melatonin in nails may provide information about the global melatonin production decreasing the sampling complexity and increasing the period under evaluation.

The main purpose of our research was to explore the potential of the determination of melatonin in nails as an alternative for the evaluation of global melatonin production. Firstly, we optimized and validated a liquid chromatography-tandem mass spectrometry (LC-MS/MS) method able to determine the low melatonin concentrations in nails. We determined the nails’ melatonin concentrations in samples collected from 84 healthy volunteers. We also explored the potential usefulness of toenails by analyzing both fingernails and toenails collected from 22 healthy volunteers. Finally, we established the biological meaning of the determination by evaluating the period in which melatonin administration was detectable in nails.

## 2. Results

### 2.1. Method Development

**Figure 1:**
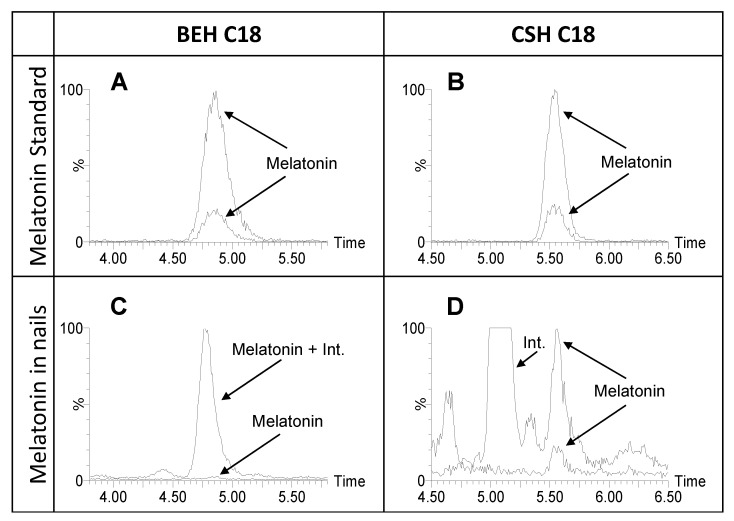
Relevance of chromatography in the LC-MS/MS detection of nail melatonin content. LC-MS/MS chromatograms for the two selected transitions of melatonin (233 > 174 and 233 > 159) in standard (250 pg mL^−1^) (**A**) and (**B**) and in a nail sample (31 fg/mg) (**C**,**D**). Chromatograms obtained by using two different chromatographic columns, BEH C18 (**A**,**C**) and CSH Phenyl-Hexyl (**B**,**D**). The time in the x axis is expressed in minutes. Abbreviation: Int.: interferent.

When using an Acquity BEH C18 column (100 mm × 2.1 mm i.d., 1.7 μm) (Waters Associates), the presence of a ubiquitous interference hindered the proper quantification of melatonin by acquiring the most abundant transition (233 > 174). The acquisition of the secondary transition (233 > 159) helped to establish the presence of that interference by comparing the ion ratio of the sample with the one of a solvent standard (Figure 1).

A more adequate separation was obtained when using an Acquity CSH C18 column (Figure 1) favoring the quantification of melatonin by the acquisition of the most abundant transition (233 > 174).

Obtaining the ion ratios between the two selected transitions (273 > 174 and 273 > 159) was required to discern the presence of endogenous interferences. The low abundance of the qualitative transition (273 > 159) hampered getting this ratio in low concentrated samples. For that reason, we sum up 10 qualitative transitions, increasing the sensitivity and allowing one to properly establish the ion ratios (Figure S1).

### 2.2. Method Validation

#### 2.2.1. Validation Using Melatonin-Free Nails

Validation results using melatonin-free nails are depicted in Table 1. The method was found to be linear in the range 6.5–830 fg/mg. The method was found to be accurate (103–115%) and precise (coefficient of variation < 8% (CV < 8%)) at four different levels. A limit of detection (LOD) of 3.5 fg/mg was established for the method.

#### 2.2.2. Validation Using Real Nail Matrix

Validation results by standard additions using real nail matrix are summarized in Table 1. Melatonin levels of the samples used for standard additions were in the concentration range 30–110 fg/mg. Both intraday (accuracy 100%, CV = 10%) and interday (accuracy 104%, CV = 13%) results confirm the suitability of the method to determine nail melatonin concentrations. The method was found to be specific (difference in ion ratios < 10% in all samples tested) with no remarkable matrix effects (recovery 105%, CV = 7%).

### 2.3. Method Application

#### 2.3.1. Establishment of Endogenous Levels of Fingernail Melatonin

**Figure 2:**
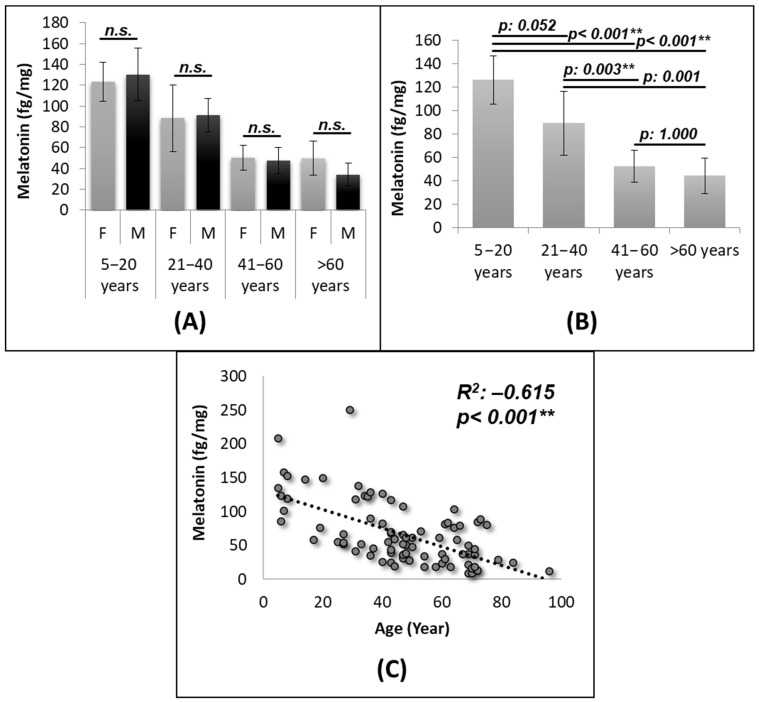
Fingernail melatonin concentrations are able to determine the gradual decrease of melatonin production over the life-span. (**A**) Gender stratified differences in the established age ranges: range 5–20, females (*n* = 6) and males (*n* = 5); range 21–40, females (*n* = 11) and males (*n* = 5); range 41–60, females (*n* = 14) and males (*n* = 15); range > 60, females (*n* = 19) and males (*n* = 9). (**B**) Differences in age ranges when considering both genders together: range 5–20 (*n* = 11); range 21–40 (*n* = 16); range 41–60 (*n* = 29); range > 60 (*n* = 28). (**C**) Correlation between fingernail melatonin concentrations and age. ** Statistically significant differences (*p* < 0.01).

The developed method was able to determine the endogenous concentrations in 84 fingernails samples (Table S1). Melatonin concentrations ranged from 58 fg/mg to 208 fg/mg for volunteers 5–20 years old (average 123 fg/mg for females and 130 fg/mg for males), from 26 fg/mg to 250 fg/mg for volunteers 31–40 years old (average 88 fg/mg for females and 91 fg/mg for males), from 18 fg/mg to 117 fg/mg for volunteers 41–60 years old (average 50 fg/mg for females and 48 fg/mg for males) and from 12 fg/mg to 89 fg/mg for volunteers > 60 years old (average 50 fg/mg for females and 34 fg/mg for males) (Figure 2A,B and Table S1). No significant differences were obtained by gender (Figure 2A), but significant differences were observed by age (Figure 2B). We also found a statistically significant decrease (Pearson coefficient −0.615, *p* < 0.001) of nail melatonin levels with age (Figure 2C) with a clear decrease until c.a. forty years of age but after concentrations seem to remain constant (Figure 2).

#### 2.3.2. Potential of Toenails for Melatonin Determination

**Figure 3:**
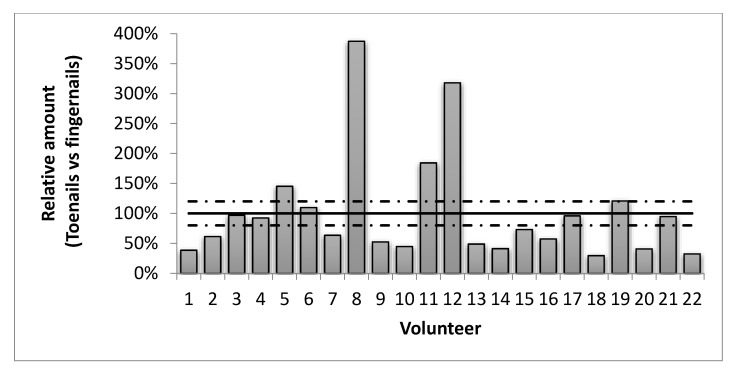
Toenail analysis failed to replace fingernails’ melatonin determination. Relative abundance of melatonin levels in toenails compared with the ones of fingernails collected at the same time from several volunteers. Dotted lines represent the 80–120% threshold required to consider the results as replaceable.

We analyzed the melatonin content in both the fingernails and the toenails collected from 22 healthy volunteers. Our results showed that comparable levels (between 80% and 120% of the fingernail result) were obtained only for six volunteers (Figure 3, Table S2). Four volunteers showed higher concentrations (150–375%) in toenails than in fingernails. Most of the volunteers (12 out of 22) exhibited lower levels (30–70%) in toenails than in fingernails. Remarkably, the correlation between melatonin production and age observed when analyzing fingernails disappeared when using data from toenails.

#### 2.3.3. Effect of Exogenous Melatonin Administration in the Fingernail’s Melatonin Levels

**Figure 4:**
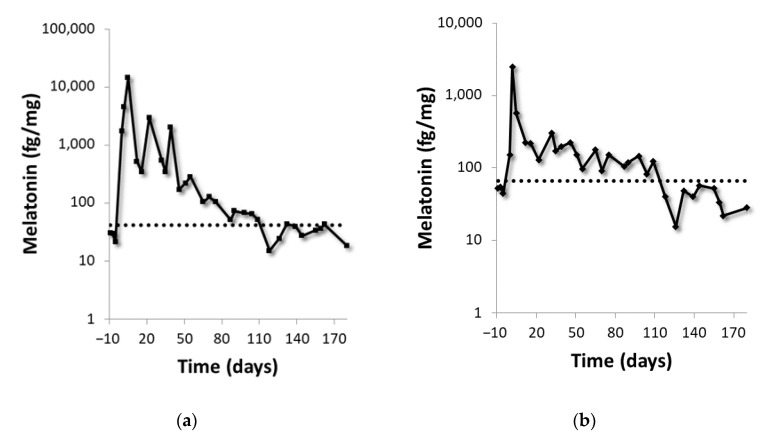
Nail melatonin content provides a weighted average of the circulating melatonin from the last 3.5 months. Changes over time in the nail’s melatonin levels (axis is represented in log scale) due to the exogenous administration of melatonin in the two volunteers: (**A**) male, (**B**) female. Dotted lines represent the endogenous levels for each volunteer calculated from basal samples (obtained between days −10 and 0).

Melatonin was evaluated in two volunteers’ fingernails during a period of 180 days after melatonin intake. The first day after melatonin administration was considered as day 1. Basal melatonin fingernail levels of volunteers were determined in three samples collected pre-administration (samples represented between day −10 and day 0) and constant values were obtained for both volunteers (Figure 4). Melatonin administration was detected in the sample collected the day after the administration with a clear increase in the nail melatonin levels (Figure 4). The melatonin levels increased and reached their maximum peak during the administration period (day 5 for the male volunteer and day 2 for the female volunteer). After reaching their maximum, nail melatonin levels gradually decreased until returning back to basal levels after 109 days.

## 3. Discussion

The potential of nail analysis for determining the global production of melatonin has been evaluated. We have developed and validated a LC-MS/MS analytical method to determine melatonin content in nails. The determination of melatonin in nails is an analytical challenge, since melatonin is present at extremely low concentrations and nails, besides its low sample availability, is a complex matrix.

The current work emphasizes the importance of chromatographic separation in order to separate an interferent ubiquitously present in nails and to provide a reliable quantification of melatonin. It also implies the need for testing the specificity of the method by evaluating the ion ratio between two transitions. This evaluation is difficult when the qualitative transition is clearly less abundant than the quantitative one (like in the case of melatonin). The strategy of adding multiple selected reaction monitoring (SRM) transitions has gained attention over recent years [33,34] for increasing sensitivity. Our results demonstrate that this strategy was useful to improve the sensitivity of the qualitative transition helping in the evaluation of the specificity of the method (Figure S2).

The analytical method was validated by two different approaches. On one hand, melatonin-free matrices were used to establish some parameters, e.g., linearity, accuracy, precision and LOD. However, since melatonin is an endogenous hormone present in each individual, actual blank samples were not available. The use of artificial melatonin-free matrices may underestimate the effect of some matrix components removed during the preparation of melatonin-free nails and for this reason, method validation was complemented by using real matrices to evaluate other parameters such as accuracy, precision and matrix effect. The results of both validation processes indicate that the developed method is suitable for the determination of nail melatonin content.

The evaluation of fingernails’ melatonin levels of 84 individuals indicate that nail melatonin content mimics the reported behavior of circulating melatonin. As expected, no significant differences were obtained by gender [35,36] (Figure 2A) but some differences were observed by age (Figure 2B). The variation found in the different age ranges (3–8 fold difference between the maximum and the minimum), specially between 5 to 40 years, are in agreement with previously reports using urinary 6-sulfatoxymelatonin marker for global melatonin production [37] (Figure 2B). Our results agree with the ones obtained by Kennaway et al. which report that melatonin levels decreased mainly in early adulthood [38]. Nail melatonin determination was also able to show the expected decline in melatonin excretion with age [37,39,40,41,42]. We found a statistically significant decrease (Pearson coefficient −0.615, *p* < 0.001) of nail melatonin levels with age (Figure 2C). The decrease of nail melatonin content observed with age reinforces the interest of using melatonin as aging biomarker [19,43] and opens the possibility of using nail melatonin content as ageing biomarker or even for the evaluation of anti-ageing therapies. In order to confirm that point, a correlation between nail melatonin content and common ageing biomarkers (oxidative stress, RedOx status, mitochondria activity, peroxisome activity, telomere length) [44,45,46] should be established. Additionally, the confirmation of these results by the analysis of a larger set of samples is advised in order to strengthen the applicability of the nail melatonin content as aging biomarker.

One of the main limitations of using fingernails for analysis is the fact that there is an important number of people with onychophagia (i.e., that bite their fingernails), particularly among the youth population [47]. For these individuals collecting fingernails is not straightforward and the analysis of toenails may be considered as alternative. Unfortunately, we observed that melatonin levels in fingernails and toenails are not comparable (Figure 3, Table S2). Thus, our results seem to advice against the use of toenails as alternative for fingernails. Several potential reasons might be behind these differences. Firstly, toenails grow 30–50% slower than fingernails [32]. This suggests that the melatonin levels found in both nail types collected at the same time may provide different information. In addition, nails are over-exposed to external factors such as sweat or shampoos. As previously reported for some metabolites in hair [48], this over-exposition might induce some degradation of melatonin in nails. Since the deposition time is longer in toenails than in fingernails, this could partially explain the commonly lower melatonin levels found in toenails. A potential poorer transference/diffusion of melatonin into toenails might also explain the lower levels found compared with fingernails although more experiments would be required to confirm this point.

In order to evaluate the biological meaning of the nail melatonin concentrations, we increased circulating levels of melatonin in two volunteers by the administration of melatonin (daily during 5 consecutive days). The main goal of this pilot study was to establish the detection window of this increase by nail analysis. The period in which the increment was detected (from hours to up to three months after the administration) indicates that melatonin is rapidly deposited into nails and quickly diffused through the nail bed. The diffusion of other substance such as methyl red sodium salt or topic drugs in nails has been previously described [49,50]. That increase together with the fact that nail melatonin levels gradually decreased to basal levels after 109 days suggested that nails melatonin determination does not capture a fixed moment in time but provides a weighted average of the circulating melatonin in the last 3–4 months.

The developed approach can be also useful to check the adherence of patients/volunteers to treatments based on melatonin administration. Melatonin metabolism in humans is rapid and its half-life varies between 10 and 60 min after the intake [51]. This fact hinders the evaluation of the adherence of patients/volunteers to melatonin treatments by plasma/saliva analysis. In comparison with these matrices, nail do not require for a fast sample collection after exogenous administration. Our results indicate that nail samples collected at any time will provide robust evidence of exogenous administration.

The proposed strategy is not exempt of limitations. Firstly, we have demonstrated that robust results are only obtained when using fingernails. Therefore, its application to patients/volunteers with onychophagia is rather limited. Additionally, expensive instruments (LC-MS/MS) seem to be required for the determination of the trace levels of nail melatonin. More research is needed in order to evaluate whether more economical approaches are able to determine these levels. Finally, this study did not evaluate the potential effect of some factors such nail dye and collection season in nail melatonin levels

## 4. Materials and Methods

### 4.1. Reagents and Chemicals

Melatonin ≥ 98% and melatonin-d4 99% were purchased in Sigma Aldrich (St Louis, MO, USA) and in Toronto Research Chemicals (North York, ON, Canada) respectively. Sodium chloride, potassium carbonate, ethyl acetate and methanol (LC gradient grade) were bought from Merck (Darmstadt, Germany) and ammonium formate (HPLC grade) from Sigma Aldrich (St Louis, MO, USA). Ultrapure water was obtained by a Milli-Q purification system (Millipore Ibérica, Barcelona, Spain).

### 4.2. Instrumentation

LC-MS/MS was used for the analysis (Waters Associates, Milford, MA, USA). The chromatography separation was achieved by an isocratic gradient at 25% (organic solvent) by using an Acquity CSH C18 column (100 mm × 2.1 mm i.d., 1.7 μm) (Waters Associates, Milford, MA, USA) at a flow rate of 300 µL min^−1^ and water and methanol as mobiles phases (both with ammonium formate (1 mM) and formic acid (0.01% *v/v*)).

The detection of melatonin was performed in the SRM mode by using the transitions of 233 > 159 (collision energy 30 V, monitored 10 times) and 233 > 174 (collision energy 10 V) for melatonin and 237 > 163 (collision energy 30 V) and 237 > 178 (collision energy 10 V) for melatonin-d4. The cone voltage was set at 20 V and the dwell times at 70 ms.

### 4.3. Sample Treatment

Nail samples were washed with 1 mL ultrapure water and vortexed for 1 min. The aqueous fraction was discarded, and nail samples were dried overnight. Then, samples were pulverized for 2 min using a ball mill (Mixer Mill MM 200 from Retsch (Haan, Germany)).

After addition of 50 µL of melatonin-d4 (500 pg mL^−1^), 30 mg of pulverized nails were extracted with 2 mL of methanol during 1 h in an ultrasounds bath (Selecta, Barcelona, Spain). Then, samples were centrifuged (3500× *g*, 5 min) and the methanolic extract was extracted and evaporated under nitrogen stream (40 °C, <15 psi) to dryness. The extract was reconstituted with 100 µL of ultrapure water and 10 µL were injected into the system.

### 4.4. Preparation of Melatonin-Free Nails

Melatonin-free matrices were prepared by extracting nail samples twice with methanol as described in Section 4.3. Methanol extracts were discarded and the dry sample was considered as melatonin-free matrix. The absence of melatonin was confirmed by LC-MS/MS analysis.

### 4.5. Method Validation

#### 4.5.1. Method Validation Using Melatonin-Free Nails

Method validation using melatonin-free nails was performed based on the criteria established on EMA regulations [52]. Briefly, linearity was evaluated by preparing calibration standards in methanol by triplicate at seven different melatonin concentrations (range 6.5–830 fg/mg).

Accuracy and precision were evaluated by the analysis of melatonin-free nails spiked at different levels (QC). The method was considered accurate when results were between 80–120% for the low QC sample and between 85–115% for medium and high QC samples [52] and precise when CV did not exceed 20% for low QC and 15% for medium and high QC [52].

The LOD was defined as the estimated providing a signal to noise ratio (S/N) of 3. The lowest limit of quantification (LLOQ) and the upper limit of quantification (ULOQ) were the lowest and the highest value described to be accurate (80–120% and 85–115% respectively) and precise (CVs < 20% and < 15% respectively).

#### 4.5.2. Method Validation Using Real Nail Matrix

Validation using real nail matrix was performed by standard addition as previously reported by our group [53,54].

Method accuracy and precision were also evaluated by performing standard addition in real nail matrices (*n* = 6) The method was considered accurate and precise if intra-assay and inter-assay accuracy ranged 80–120% and CVs were below 20%.

Matrix effect of real nails was calculated in terms of recovery by comparing the slopes obtained in the standard addition and the solvent standards. The recovery was calculated as “Recovery = (slope standard addition) / (slope in solvent)×100” and was considered satisfactory in the range of 80–120% and CV below 20%.

Specificity was tested in real nails (*n* = 5) by comparing the ion ratio between the two selected transitions obtained in human samples with the one obtained in methanolic solutions (*n* = 7). Differences below 20% in ion ratio comparison were considered as indicative of the absence of significant interferences in real nail samples.

### 4.6. Method Application

The applicability of the method was evaluated by 3 different experiments. Firstly, 84 fingernails’ samples selected from males and females and at different range ages (described in Section 4.7.2) were evaluated to establish the endogenous melatonin levels in fingernails. Secondly, 22 volunteers that provide fingernails also provided toenails’ samples and a comparison between both nail samples was performed to evaluate the possibility to use toenails instead of fingernails. Then, two volunteers were administered by melatonin during 5 days and the effect of melatonin administration in melatonin fingernails levels was studied.

### 4.7. Samples

The free margin of nail samples was collected by scissor cutting and stored at room temperature until analysis. The study was performed according to the principles outlined by the Helsinki Declaration and informed consent was obtained from all volunteers.

#### 4.7.1. Method Validation

Nail samples from 6 healthy volunteers (ages from 5 to 60, not taking exogenous melatonin) were collected for 2 months and combined (one sample per volunteer).

#### 4.7.2. Method Application

##### Endogenous Melatonin Levels in Fingernails

For establishment of endogenous levels, fingernail samples were collected from 84 healthy volunteers (50 females (ages 5–96 years old), 34 males (ages 5–79 years old)).

##### Comparison of Melatonin Levels between Fingernails and Toenails

Twenty-two of the aforementioned volunteers also provided toenail samples (13 females and 9 males, ages 5–75 years old).

##### Effect of Melatonin Administration in Melatonin Fingernails’ Levels

In order to have a first estimation of the detection time window, a pilot study was performed. Two healthy volunteers (one female, 44 years old; one male, 44 years old) orally took one melatonin tablet (1.9 mg tablets from ESI melatonin Pure, ESI srl, Italy) daily during 5 consecutive days (administration time around 2 h before going bed). Nail samples from 3–4 fingers were collected as described in Figure S2. Briefly, 3 samples were collected before administration to establish basal levels and subsequently one sample was collected every 2–18 days for 180 days starting the day after the first administration.

### 4.8. Data Analysis

Quantification of melatonin was performed using MassLynx software (version 4.1) from Waters Associates, Milford, MA, USA.

The statistical analysis was performed by the SPSS software (version 18.0; IBM, Armonk, New York, NY, USA). The influence of gender and age in the fingernail’s melatonin was evaluated by a two-way ANOVA and the influence of age in the fingernail’s melatonin was determined by a one-way ANOVA with a Bonferroni post-hoc test (significant levels was considered when *p* < 0.05). The Pearson’s correlation with a two-tailed test was performed to assess the correlations between fingernails’ melatonin and age (significant level at *p* < 0.05).

In order to estimate the effect of melatonin administration, the detection window was estimated by evaluating the period in which the melatonin levels exceeded the basal ones. Briefly, an individual threshold value for melatonin was calculated for each volunteer as Threshold = (mean melatonin in basal samples)+ 3×(standard deviation in basal samples). The detection window for each volunteer was defined as the period in which values exceeded the individual threshold [55,56].

## 5. Conclusions

The potential of nail analysis for determining the global production of melatonin has been evaluated. Our results indicate that fingernail melatonin content mimics the expected behavior of circulating melatonin. Therefore, the determination of nail melatonin content may be a promising tool for obtaining information about the global production of melatonin and pineal gland status. This strategy might reveal new insights into some pathological states with disturbances in melatonin production.

## Figures and Tables

**Table 1:** Method validation parameters in melatonin-free matrix and real nail matrix. Calculated melatonin-free matrix parameters were linearity (in terms of weighting and range), LLOQ, LQC, MQC, HQC and LOD (in terms of accuracy and CV). Calculated real nail matrix parameters were within-run and total accuracy and CV; matrix effect (in terms of recovery and CV); the endogenous range of selected samples’ melatonin; and specificity of the method by comparing standard and samples. Units of range, level, LOD and concentration are fg/mg.

Melatonin-Free Matrix (CDER Regulations)						
**Linearity**	**Weight.**	**Range (fg/mg)**				
	1/x	6.5–830				
	**LLOQ**	**LQC**	**MQC**	**HQC**	**LOD**	
Level	6.5	13	0.104	830	3.5	
Acc. (%)	113	115	104	103	-	
CV (%)	8	6	4	4	-	
**Real nail matrix (Standard additions)**						
	**Within-run (*n* = 6)**	**Total (*n* = 12)**		**M.E. (*n* = 6)**		**Endog Range**
Acc. (%)	100	104	Rec. (%)	105	Conc.	30–110
CV (%)	10	13	CV (%)	7	(fg/mg)	
**Specificity**						
	**Standard**	**Sample 1**	**Sample 2**	**Sample 3**	**Sample 4**	**Sample 5**
Q/q ratio	0.22	0.23	0.21	0.24	0.23	0.22

Abbreviations: CDER: Center for Drug Evaluation and Research; Weight: weighting; LLOQ: lowest limit of quantification; LQC: lowest QC; MQC: medium QC; HQC: highest QC; LOD: limit of detection; Acc.: accuracy; CV: coefficient of variation; M.E.: matrix effect; Endog Range: endogenous range; Rec.: recovery; Conc: concentration; Q/q: ion ratio between quantitative transition (233 > 174) and qualitative transition (233 > 159).

## Data Availability

The data presented in this study are available on request from the corresponding author.

## Supplementary Materials

The following are available online at https://www.mdpi.com/1422-0067/22/2/921/s1, Figure S1: Chromatogram representation of SRM transitions of melatonin in a real nail sample (18 fg/mg). (A) Melatonin-d4, (B) Melatonin (273 > 174 transition, quantitative), (C) Melatonin (summation of 273 > 159 transition, qualitative) and (D) Melatonin (single 273 > 159 transition, qualitative), Figure S2: General overview of the design and schedule of the study of melatonin administration. (A) Distribution of the different collected (a) nails collected from both little fingers and both ring fingers (4 nails), (b) nails collected from right thumb and left index finger and middle finger (3 nails) and (c) nails collected from left thumb and right index finger and middle finger (3 nails); (B) Schedule of samples collection (in orange sample collection (a), in blue sample collection (b) and in red sample collection (c)). In dots, days that exogenous melatonin was administered to volunteers are highlighted, Table S1: Melatonin levels (in fg/mg) in the selected population. Abbreviations: F: female and M: male, Table S2: Melatonin levels (in fg/mg) in fingernails and toenails. Abbreviations: F: female and M: male.

## Abbreviations

DLMO	Dim light melatonin onset
LC-MS/MS	Liquid chromatography tandem mass spectrometry
LOD	Limit of detection
CV	Coefficient of variation
CDER	Center for Drug Evaluation and Research
SRM	Selected reaction monitoring
QC	Melatonin-free nails spiked at different levels
S/N	Signal to noise ratio
