# Supplementary material for: Nail Melatonin Content: A Suitable Non-Invasive Marker of Melatonin Production
Source: Int J Mol Sci. 2021 Jan 18;22(2):921. doi: 10.3390/ijms22020921 (PMC7831915; doi:10.3390/ijms22020921)
Supplement: Supplementary file 1 [file ijms-22-00921-s001.pdf]

Nail Melatonin Content: A Suitable Non-Invasive Marker of Melatonin Production

Alex Gomez-Gomez, Blanca Montero-San Martin, Noemí Haro and Oscar J Pozo

Supplemental Information

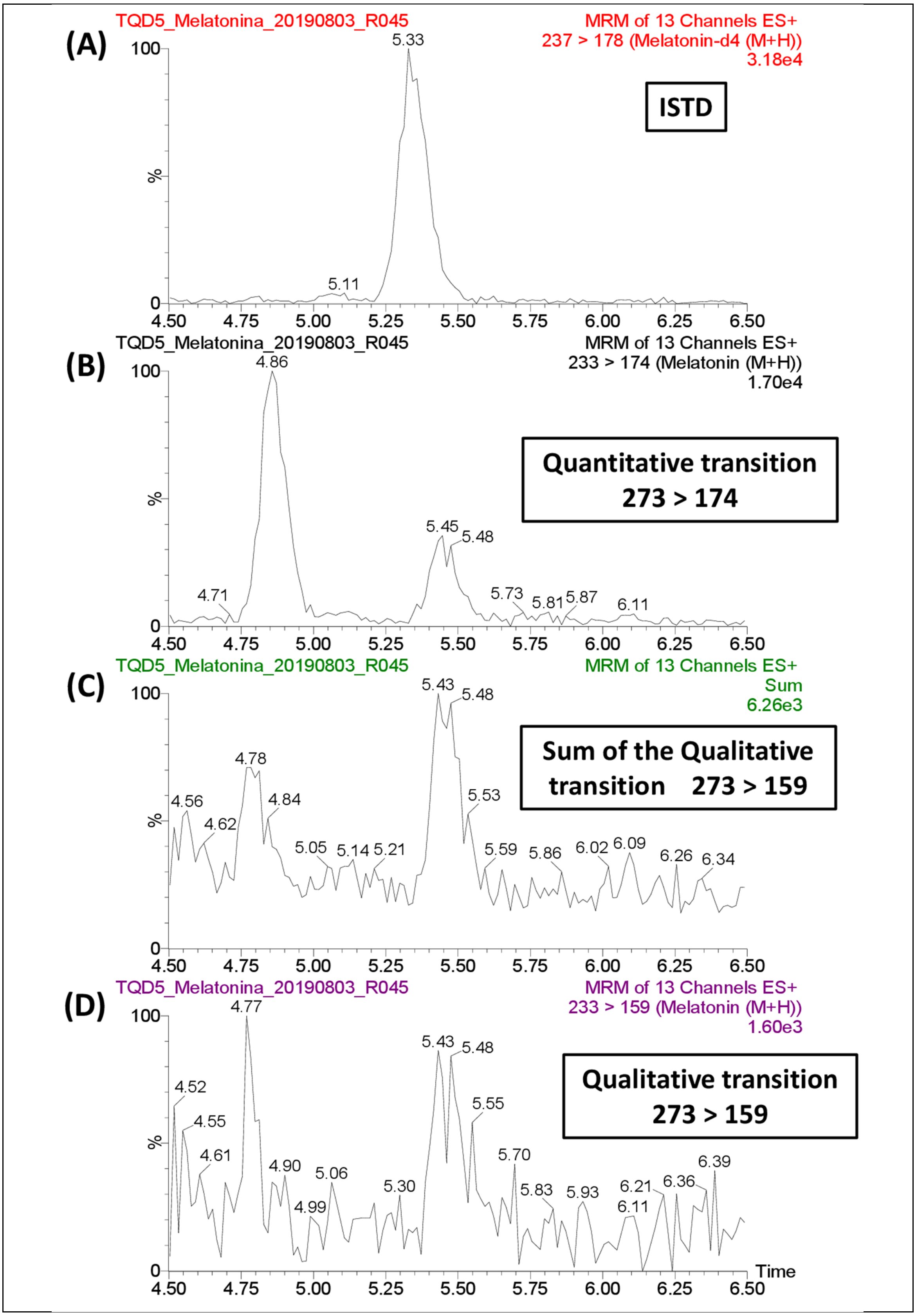

Figure S1. Chromatogram representation of SRM transitions of melatonin in a real nail sample (18 fg/mg). (A) Melatonin-d4, (B) Melatonin (273 > 174 transition, quantitative), (C) Melatonin (summation of 273 > 159 transition, qualitative) and (D) Melatonin (single 273 > 159 transition, qualitative).

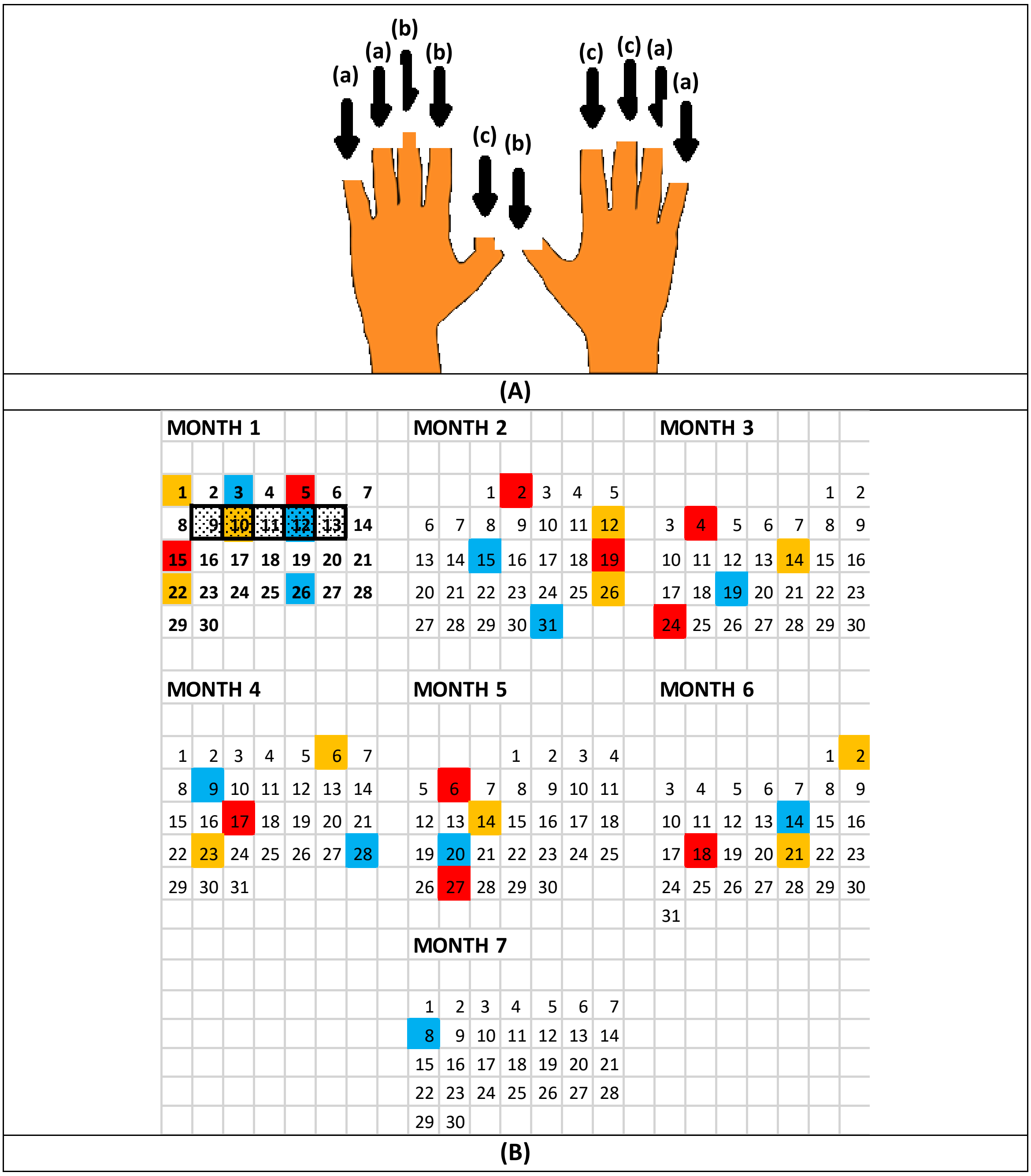

Figure S2. General overview of the design and schedule of the study of melatonin administration. (A) Distribution of the different collected (a) nails collected from both little fingers and both ring fingers (4 nails), (b) nails collected from right thumb and left index finger and middle finger (3 nails) and (c) nails collected from left thumb and right index finger and middle finger (3 nails); (B) Schedule of samples collection (in orange sample collection (a), in blue sample collection (b) and in red sample collection (c)). In dots, days that exogenous melatonin was administered to volunteers are highlighted.

Table S1. Melatonin levels (in fg/mg) in the selected population. Abbreviations: F: female and M: male.

Vol.	Age	Sex	Melatonin	Vol.	Age	Sex	Melatonin	Vol.	Age	Sex	Melatonin
**1**	5	F	134	**29**	50	F	61	**57**	31	M	118
**2**	6	F	86	**30**	54	F	18	**58**	32	M	138
**3**	6	F	123	**31**	59	F	62	**59**	33	M	52
**4**	7	F	158	**32**	60	F	23	**60**	36	M	89
**5**	8	F	153	**33**	60	F	38	**61**	40	M	82
**6**	17	F	58	**34**	61	F	82	**62**	42	M	55
**7**	20	F	149	**35**	62	F	83	**63**	43	M	44
**8**	25	F	55	**36**	64	F	76	**64**	43	M	117
**9**	27	F	52	**37**	64	F	103	**65**	44	M	20
**10**	27	F	54	**38**	66	F	79	**66**	44	M	60
**11**	29	F	250	**39**	69	F	9	**67**	47	M	31
**12**	31	F	42	**40**	69	F	38	**68**	47	M	36
**13**	34	F	123	**41**	69	F	50	**69**	47	M	52
**14**	35	F	122	**42**	70	F	9	**70**	48	M	38
**15**	36	F	35	**43**	71	F	15	**71**	48	M	61
**16**	36	F	128	**44**	71	F	36	**72**	49	M	28
**17**	37	F	45	**45**	71	F	45	**73**	53	M	71
**18**	40	F	26	**46**	72	F	13	**74**	54	M	34
**19**	40	F	127	**47**	72	F	85	**75**	58	M	18
**20**	43	F	25	**48**	73	F	89	**76**	61	M	30
**21**	43	F	39	**49**	84	F	25	**77**	63	M	19
**22**	43	F	67	**50**	96	F	12	**78**	65	M	58
**23**	43	F	69	**51**	5	M	208	**79**	67	M	37
**24**	47	F	34	**52**	7	M	102	**80**	69	M	21
**25**	47	F	65	**53**	8	M	119	**81**	70	M	16
**26**	47	F	107	**54**	14	M	147	**82**	71	M	19
**27**	48	F	51	**55**	19	M	76	**83**	75	M	80
**28**	50	F	47	**56**	27	M	67	**84**	79	M	29

Table S2. Melatonin levels (in fg/mg) in fingernails and toenails. Abbreviations: F: female and M: male.

Vol.	Age	Sex	Melatonin (fg/mg)	
			Fingernails	Toenails
1	5	F	134	52
2	6	F	86	53
3	6	F	123	119
4	8	F	153	141
5	27	F	52	75
6	40	F	127	139
7	43	F	67	42
8	47	F	34	131
9	47	F	107	56
10	50	F	47	21
11	58	F	67	47
12	59	F	62	196
13	71	F	45	22
14	27	M	67	28
15	32	M	138	101
16	42	M	55	32
17	43	M	117	112
18	44	M	60	18
19	47	M	31	37
20	48	M	24	10
21	61	M	30	28
22	75	M	80	26

Abbreviations: Vol.: Volunteer.
